# Vascular Endothelial Growth Factor Gene Polymorphism (rs2010963) and Its Receptor, Kinase Insert Domain-Containing Receptor Gene Polymorphism (rs2071559), and Markers of Carotid Atherosclerosis in Patients with Type 2 Diabetes Mellitus

**DOI:** 10.1155/2016/1482194

**Published:** 2015-12-31

**Authors:** Sebastjan Merlo, Jovana Nikolajević Starčević, Sara Mankoč, Marija Šantl Letonja, Andreja Cokan Vujkovac, Marjeta Zorc, Daniel Petrovič

**Affiliations:** ^1^Institute of Oncology Ljubljana, Zaloška 2, Sl-1000 Ljubljana, Slovenia; ^2^Institute of Histology and Embryology, Faculty of Medicine, University in Ljubljana, Vrazov trg 2, Sl-1000 Ljubljana, Slovenia; ^3^General Hospital Rakičan, Ulica dr. Vrbnjaka 6, Sl-9000 Murska Sobota, Slovenia; ^4^General Hospital Slovenj Gradec, Gosposvetska Cesta 1, Sl-2380 Slovenj Gradec, Slovenia

## Abstract

*Background*. The current study was designed to reveal possible associations between the polymorphisms of the vascular endothelial growth factor (VEGF) gene (rs2010963) and its receptor, kinase insert domain-containing receptor (KDR) gene polymorphism (rs2071559), and markers of carotid atherosclerosis in patients with type 2 diabetes mellitus (T2DM).* Patients and Methods*. 595 T2DM subjects and 200 control subjects were enrolled. The carotid intima-media thickness (CIMT) and plaque characteristics (presence and structure) were assessed ultrasonographically. Biochemical analyses were performed using standard biochemical methods. Genotyping of VEGF/KDR polymorphisms (rs2010963, rs2071559) was performed using KASPar assays.* Results*. Genotype distributions and allele frequencies of the VEGF/KDR polymorphisms (rs2010963, rs2071559) were not statistically significantly different between diabetic patients and controls. In our study, we demonstrated an association between the rs2071559 of KDR and either CIMT or the sum of plaque thickness in subjects with T2DM. We did not, however, demonstrate any association between the tested polymorphism of VEGF (rs2010963) and either CIMT, the sum of plaque thickness, the number of involved segments, hsCRP, the presence of carotid plaques, or the presence of unstable carotid plaques.* Conclusions*. In the present study, we demonstrated minor effect of the rs2071559 of KDR on markers of carotid atherosclerosis in subjects with T2DM.

## 1. Introduction 

Type 2 diabetes mellitus (T2DM) is considered a major epidemic of this century. It is estimated that its prevalence will increase worldwide from 371 million people in 2013 to 552 million people in 2030 [1]. T2DM is associated with accelerated progression of atherosclerosis, the major cause of vascular complications leading to increased morbidity and mortality [2].

Chronic, low-grade inflammation has been demonstrated to be involved in the pathogenesis of atherosclerosis in subjects at high risk to develop cardiovascular disease [3–7]. Among immune cells infiltrating atherosclerotic lesions, polymorphonuclear neutrophil leukocytes with their products were reported to have an important role in the development and progression of atherosclerosis [8–11]. Marino and coworkers have recently reported that both circulating and intraplaque polymorphonuclear neutrophil leukocytes from subjects with carotid atherosclerosis are active producers of different inflammatory mediators including the vascular endothelial growth factor (VEGF) [11].

Several environmental and genetic factors (i.e., hypoxia, hyperglycemia, oxidative stress, ischemia, and gene polymorphisms of VEGF) influence plasma VEGF levels [12–16]. Among several polymorphisms of the VEGF gene, the rs2010963 (−634C/G polymorphism of the VEGF gene) and few others were reported to affect serum VEGF levels [13–15]. Moreover, rs2010963 was demonstrated to be associated with several disorders, such as diabetic retinopathy, diabetic nephropathy, myocardial infarction, and impaired prognosis in patients with chronic heart failure [13–15, 17]. Despite these findings, however, data about VEGF polymorphisms and their possible association with carotid atherosclerosis in patients with diabetes mellitus are limited [18–20]. Additionally, CIMT is highly heritable and associated with stroke and myocardial infarction, making it a promising quantitative intermediate phenotype for genetic studies of vascular disease [21].

The present study was thus designed to investigate the association between polymorphisms of the VEGF gene (rs2010963) and the KDR gene (rs2071559) and markers of carotid atherosclerosis (such as carotid intima-media thickness (CIMT), the number of affected segments of carotid arteries, and the sum of plaques thickness) in patients with T2DM.

## 2. Material and Methods

The study protocol was approved by the Slovene Medical Ethics Committee in September 2010 (Protocol number 128/09/2010). After an informed consent for the participation in the study was obtained, a detailed interview was made.

This cross-sectional study included 595 subjects with T2DM and 200 subjects without T2DM (control group). They were selected among patients admitted to the diabetes outpatient clinics of the General Hospitals Murska Sobota and Slovenj Gradec, Slovenia. Subjects in the control group were not allowed to have T2DM, and they were the staff of the General Hospital Murska Sobota. Subjects with T2DM and control subjects were excluded if they had homozygous familial hypercholesterolaemia or a previous cardiovascular event such as myocardial infarction or a cerebral stroke.

All ultrasound examinations were performed by two experienced doctors blinded to the participants' diabetes status. The CIMT, defined as the distance from the leading edge of the lumen-intima interface to the leading edge of the media-adventitia interface, was measured, as described previously [22]. Plaques were defined as a focal intima-media thickening and divided into 5 types according to their echogenic/echolucent characteristics, as previously described [22]. The interobserver reliability for carotid plaque characterization was found to be substantial (*κ* = 0.64, *p* < 0.001).

The genomic DNA was extracted from 100 *μ*L of whole blood using a FlexiGene DNA isolation kit, in accordance with the recommended protocol (Qiagene GmbH, Hilden, Germany).

For VEGF rs2010963 polymorphism competitive allele specific PCR (KASP) was conducted on an ABI Step-One System (Applied Biosystems, Foster City, CA). The reaction mixture (5 *μ*L) contained 2.5 *μ*L 2x KASPar reaction Mix (v3), 0.07 *μ*L Assay Mix, 1.43 *μ*L of distilled water Dnase/RNase-free (Gibco, Invitrogen Life Technologies), and 10 ng of extracted genomic DNA (1 *μ*L). Thermal cycling employed the following conditions: hot-start enzyme activation (15 min at 94°C), denaturation (20 sec at 94°C) followed by 10 cycles of touchdown over 65–57°C for 60 sec (dropping 0.8°C per cycle), and final 26 cycles (20 sec at 94°C and 60 sec at annealing temperature 57°C). For rs2071559 (KDR) everything was the same with the exception of thermal conditions. Hot-start enzyme activation (15 min at 94°C) and denaturation (20 sec at 94°C) were followed by 15 cycles of touchdown over 55–65°C for 60 sec (dropping 0.8°C per cycle) and final 26 cycles (20 sec at 93°C and 60 sec at annealing temperature 58°C).

In addition, the fasting serum VEGF levels were analyzed in 70 subjects with T2DM and in 33 subjects with T2DM. For the determination of the fasting serum VEGF concentration (isoform VEGF 165), a solid phase sandwich ELISA using two kinds of high specific antibodies (hVEGF Assay Kit, IBL Co., Ltd. Aramachi, Takasaki-shi, Gunma, Japan) was used. The respective CV (%) were between 3 and 5.5 for interassay measurements and between 2.6 and 5.3 for intra-assay measurements.

Continuous variables are expressed as means ± standard deviations. Continuous clinical data were compared using unpaired Student's *t*-test or analysis of variance (ANOVA). The Pearson *χ* ^2^ test was used to compare discrete variables. A two-tailed *p* value of less than 0.05 was considered statistically significant. A statistical analysis was performed using the SPSS program for Windows version 21 (SPSS Inc., Chicago, Ilinois, USA).

## 3. Results

Patients with T2DM were older, had a greater waist circumference, and had higher fasting glucose and HbA1c levels compared to controls, whereas there were no differences in BMI and systolic and diastolic blood pressure between patients with T2DM and control subjects (Table 1). Patients with T2DM had lower total, HDL, and LDL cholesterol levels and a higher triglyceride level compared to controls (Table 1). Plasma levels of inflammatory markers (i.e., hs-CRP and fibrinogen) were higher in patients with T2DM compared to controls (Table 1). Additionally, there was higher percentage of men, statin therapy, and antihypertensive therapy and lower percentage of smokers in T2DM group compared to control group (Table 1).

The genotype distributions in both patients with T2DM and controls were in Hardy-Weinberg equilibrium for both VEGF gene polymorphisms [rs2010963: T2DM (genotype frequencies: CC genotype 8.7%, CG genotype 47.1%, and GG genotype 44.2%; *χ* ^2^ = 3.48; *p* = 0.06) and controls (genotype frequencies: CC genotype 9%, CG genotype 48%, and GG genotype 43%; *χ* ^2^ = 1.46; *p* = 0.22)]. The genotype distributions in both patients with T2DM and controls were in Hardy-Weinberg equilibrium for the KDR gene polymorphism [rs2071559: T2DM (genotype frequencies: CC genotype 22.0%, CT genotype 51.9%, and TT genotype 26.1%; *χ* ^2^ = 0.97; *p* = 0.33) and controls (genotype frequencies: CC genotype 30.0%, CT genotype 48.0%, and TT genotype 22.0%; *χ* ^2^ = 0.63; *p* = 0.23)]. No statistically significant differences in the VEGF rs2010963 and KDR rs2071559 genotype distribution frequencies were observed between T2DM patients and controls.

The observed minor allele frequency (MAF) distributions were mostly in agreement with the 1000 Genomes Project data in the European population. The C allele frequency of the VEGF rs2010963 showed no significant difference (*p* = 0.79) between patients with T2DM and controls (32.3% versus 33%). However, the C allele frequency of the KDR rs2071559 polymorphism was significantly lower (*p* = 0.04) in T2DM subjects as compared to the controls (49% versus 54%).

Higher VEGF serum levels were demonstrated in subjects with T2DM with the CC genotype (rs2010963) compared to those with other (CG + GG) genotypes (Table 2). Moreover, higher VEGF serum levels were found in subjects with the CC genotype (rs2071559) compared to those with other (CT + TT) genotypes (Table 2).

The comparison of atherosclerosis parameters was performed with regard to different genotypes of the VEGF polymorphism (rs2010963) upon enrolment. In our study, we did not demonstrate any association between the rs2010963 and either CIMT, the sum of plaque thickness, the number of involved segments, hsCRP or the presence of carotid plaques, or the presence of unstable carotid plaques (Tables 3 and 4). We did, however, demonstrate an association between the rs2071559 and either CIMT or the sum of plaque thickness in subjects with T2DM (Table 3).

## 4. Discussion

In our study, we demonstrated an association between the rs2071559 of KDR and CIMT in subjects with T2DM, whereas we did not demonstrate an association between tested polymorphism of VEGF (rs2010963) and CIMT. Variations in the VEGF gene were reported to be weakly associated with CIMT [19]. None of the single genotyped polymorphisms (−2578A>C rs699947, −634C>G rs2010963, and +936C>T rs3025039) were significantly associated with overall IMT in the study reported by Kangas-Kontio and coworkers [19]. The haplotype CCC, however, was associated with higher overall CIMT in women and the haplotype CCT with higher CIMT in the internal carotid artery in men [19].

Additionally, we also demonstrated an association between the rs2071559 of KDR and the sum of plaque thickness in subjects with T2DM, whereas no association between tested polymorphism of VEGF (rs2010963) and markers of carotid atherosclerosis was demonstrated. The rs2010963 polymorphism of the VEGF gene was not demonstrated to exert a significant influence on the risk of subclinical atherosclerosis manifested by the presence of endothelial dysfunction by brachial artery reactivity and increased CIMT in a series of patients with rheumatoid arthritis [23]. Contrary, the importance of VEGF and its receptor (VEGFR1) was reported by Russell and coworkers [24]. They analyzed 34 intact carotid endarterectomy specimens and compared histologically stable and unstable plaques. In unstable plaques (cap rupture/thinning) increased VEGF and receptor (VEGFR1) staining as well as increased microvessel density was demonstrated in comparison with stable carotid plaques [24]. Additionally, Marino and coworkers have recently reported that both circulating and intraplaque polymorphonuclear neutrophils (PMN) from subjects with carotid atherosclerosis are active producers of VEGF, IL-8, and elastase [11]. Moreover, an evidence is provided that these PMN have an increased ability to produce VEGF (at mRNA levels) in comparison to cells from healthy subjects. Additionally, increased VEGF mRNA occurs in both intraplaque and circulating PMN, at rest as well as after stimulation, suggesting that such functional changes are systemic and not limited to cells infiltrating the vascular wall [11]. In contrast to these findings, we did not demonstrate an effect of VEGF/KDR polymorphisms on the presence of either plaques or unstable plaques, since no difference in genotype distribution was present.

In our study, the effect of either rs2071559 of KDR or rs2010963 on VEGF serum levels was demonstrated. These findings are in accordance with our previous studies in which subjects with recent MI history (up to 9 months after MI) were enrolled [13, 16, 25]. Moreover, increased plasma VEGF levels demonstrated in the stable phase after MI correlated with inflammation cytokines (IL-8 and IL-6), but not with atherosclerotic burden [25].

In contrast to the minor effect of the rs2071559 of KDR and the absence of the rs2010963 of the VEGF, an association of either rs2071559 or rs2010963 with MI has recently been reported in Caucasians with T2DM [13, 16, 24]. Our present findings and previous reports are additional evidence that markers of carotid atherosclerosis and atherothrombotic events (i.e., MI) are most probably not regulated via similar genetical/biological mechanisms.

To conclude, in our study we demonstrated a minor effect of the rs2071559 of KDR on markers of carotid atherosclerosis (CIMT, sum of plaque thickness) in subjects with T2DM, whereas we failed to demonstrate an effect of tested polymorphism of the VEGF gene (rs2010963) on markers of carotid atherosclerosis.

## Figures and Tables

**Table 1:** Baseline characteristics of subjects with T2DM and subjects without T2DM (control group).

	Subjects with T2DM *n* = 595	Control group *n* = 200	*p*
Age (years)	62.39 ± 9.61	60.07 ± 9.18	0.008
Male sex (%)	338 (56.8)	92 (46.0)	0.008
Diabetes duration (years)	11.25 ± 7.88	—	—
Cigarette smoking (%)	53 (8.91)	34 (17.0)	0.002
Waist circumference (cm)	108.65 ± 12.88	93.31 ± 13.18	<0.001
BMI (kg/m^2^)	31.00 ± 4.74	27.90 ± 4.42	0.16
SBP (mm Hg)	147.1 ± 19.80	143.3 ± 16.6	0.86
DBP (mm Hg)	85.78 ± 11.60	84.7 ± 11.6	0.19
Fasting glucose (mmol/L)	8.04 ± 2.57	5.27 ± 0.87	<0.001
HbA1c (%)	7.89 ± 3.56	4.79 ± 0.29	<0.001
Total cholesterol (mmol/L)	4.70 ± 1.18	5.36 ± 1.08	<0.001
HDL cholesterol (mmol/L)	1.20 ± 0.35	1.43 ± 0.37	<0.001
LDL cholesterol (mmol/L)	2.63 ± 0.94	3.24 ± 0.98	<0.001
Triglycerides (mmol/L)	1.9 (1.2–2.7)	1.3 (0.9–1.9)	<0.001
hs-CRP (mg/L)	3.5 ± 1.18	2.2 ± 1.18	<0.001
CIMT (*μ*m)	958 ± 194	890 ± 212	0.007
Statin therapy (%)	375 (63.0)	62 (31.0)	<0.001
Antihypertensive agents (%)	499 (83.9)	58 (29%)	<0.001

Continuous variables were expressed as means ± standard deviations when normally distributed and as median (interquartile range) when asymmetrically distributed. Categorical variables were expressed as frequency (percentage). BMI: body mass index; SBP: systolic blood pressure; DBP: diastolic blood pressure; HbA1c: glycated haemoglobin; hs-CRP: high sensitivity C-reactive protein.

**Table 2:** VEGF serum levels in subjects with and without T2DM with regard to the rs2010963 and rs2071559 genotypes.

rs2010963	Mean (95% CI)		*p*	Linear trend analysis	
	CC (52)	CG + GG (543)		*F*	*p*
VEGF (ng/L)	63.5 ± 29.2	46.1 ± 22.3	**<0.01**	3.22	**0.03**

rs2071559	Mean (95% CI)		*p*	Linear trend analysis	
	CC (131)	CT + TT (464)		*F*	*p*

VEGF (ng/L)	69.4 ± 25.1	40.9 ± 28.3	**<0.01**	3.70	**0.02**

**Table 3:** Comparison of markers of carotid atherosclerosis (CIMT, sum of plaque thickness, and number of involved segments) in subjects with T2DM at the beginning of the study with regard to the rs2010963 and rs2071559 genotypes.

rs2010963	Mean (95% CI)			*p*	Linear trend analysis	
	CC (52)	CG (280)	GG (263)		*F*	*p*
CIMT (*μ*m)	1045 ± 192 (969–1121)	996 ± 210 (964–1026)	1026 ± 210 (995–1058)	0.27	2.29	0.13
Sum of plaque thickness (mm)	7.58 ± 4.52 (5.67–9.49)	7.79 ± 4.28 (7.09–8.48)	8.11 ± 4.73 (7.35–8.88)	0.76	0.009	0.93
Number of involved segments	2.67 ± 1.51 (2.07–3.26)	2.48 ± 1.70 (2.24–2.73)	2.54 ± 1.60 (2.31–2.77)	0.84	0.34	0.56

rs2071559	Mean (95% CI)			*p*	Linear trend analysis	
	CC (131)	TC (309)	TT (155)		*F*	*p*

CIMT (*μ*m)	1053 ± 186 (1012–1092)	1029 ± 200 (987–1070)	988 ± 219 (958–1019)	**0.04**	5.64	**0.04**
Sum of plaque thickness (mm)	8.81 ± 4.30 (7.83–9.78)	8.27 ± 4.50 (7.26–9.29)	7.31 ± 4.48 (6.61–8.00)	**0.03**	5.91	**0.02**
Number of involved segments	2.87 ± 1.41 (0.94–1.65)	2.38 ± 1.60 (0.95–1.73)	2.24 ± 1.70 (0.98–1.46)	0.64	0.22	0.83

**Table 4:** Comparison of markers of carotid atherosclerosis (presence of carotid plaques, presence of unstable plaques) in subjects with T2DM at the beginning of the study with regard to the rs2010963 and rs2071559 genotypes.

	rs2010963				rs2071559			
	CC (52)	CG (280)	GG (263)	*p*	CC (131)	TC (309)	TT (155)	*p*
Presence of carotid plaques *n* (%)	46 (88.5)	229 (81.8)	223 (84.7)		117 (89.3)	250 (80.9)	133 (85.8)	
OR (95% CI)	*∗*	0.68 (0.46–2.57)	0.72 (0.41–1.26)	0.45	*∗*	0.57 (0.53–2.06)	0.68 (0.34–1.34)	0.15
*p* ^†^	—	0.70	0.24		—	0.59	0.26	

Presence of unstable carotid plaques *n* (%)	27 (51.9)	143 (51.1)	121 (46.0)		69 (52.7)	142 (46.0)	77 (49.7)	
OR (95% CI)	*∗*	1.09 (0.22–3.66)	0.97 (0.38–2.50)	0.45	*∗*	0.55 (0.14–2.18)	0.67 (0.20–2.26)	0.42
*p* ^†^	—	0.56	0.59		—	0.39	0.51	

^*∗*^Reference genotype is CC.

^†^ *p* value for logistic regression analysis.
